# Mesangial angiogenesis and interstitial eosinophilic infiltration in diabetic nephropathy are associated with elevated CD248 expression

**DOI:** 10.1080/0886022X.2025.2510552

**Published:** 2025-06-01

**Authors:** Xiangmeng Li, Jiao Zhang, Ying Wang, Tianyu Yu, Shimin Jiang, Yan Gao, Haisong Zhang, Wenge Li

**Affiliations:** a Department of Nephrology, China-Japan Friendship Hospital, Beijing, China; b China-Japan Friendship Hospital, Chinese Academy of Medical Sciences & Peking Union Medical College, Beijing, China; c Key Laboratory of Bone Metabolism and Physiology in Chronic Kidney Disease of Hebei Province, Affiliated Hospital of Hebei University, Baoding, Hebei Province, China; d Department of Nephrology, Affiliated Hospital of Hebei University, Baoding, Hebei Province, China; e Beijing University of Chinese Medicine, Beijing, China

**Keywords:** Diabetic nephropathy, CD248, angiogenesis, eosinophilic infiltration, immune-inflammatory

## Abstract

**Objectives:**

To investigate the factors associated with angiogenesis within the glomerular mesangial area and interstitial eosinophilic infiltration in diabetic nephropathy (DN).

**Methods:**

The NCBI database identified differentially expressed genes (DEGs) in DN patients linked to angiogenesis and inflammation. Bioinformatics analyzed these genes and mechanisms. *In vivo* (DN patients and db/db mice) and *in vitro* experiments explored glomerular mesangial angiogenesis and interstitial eosinophilic infiltration mechanisms.

**Results:**

Twenty-five independent DEGs associated with DN were identified, and CD248 was associated with vessel formation and inflammatory cells. Biological analysis suggested CD248 mainly promoted vessel formation and eosinophilic infiltration *via* VEGFC and CCL-5, respectively. In DN patients, neovascularization with CD31-positive endothelial cells was observed in the mesangial regions, which was accompanied by increased expression of CD248 and VEGFC. Eosinophilic infiltration was observed in the renal interstitium, and the degree of eosinophilic infiltration was positively correlated with the intensity of CD248 expression. Serial section analysis revealed that areas with increased eosinophilic infiltration exhibited stronger infiltration of CD3-positive cells and elevated CCL-5 expression. Similar findings were discovered in the db/db mice, with WB results demonstrating higher expression levels of CD248, CCL-5, and VEGFC in the renal tissues of db/db mice compared with m/m mice. *In vitro*, CD248 expression is low in mesangial cells, but increased under high-glucose/LPS. CD248 siRNA reduced high-glucose/LPS-induced VEGFC/CCL-5.

**Conclusion:**

In DN, CD248 may contribute to mesangial angiogenesis and renal interstitial eosinophilic infiltration; these pathological processes may be associated with the elevated expressions of VEGFC and CCL-5, respectively.

## Graphical Abstract

**Figure:**
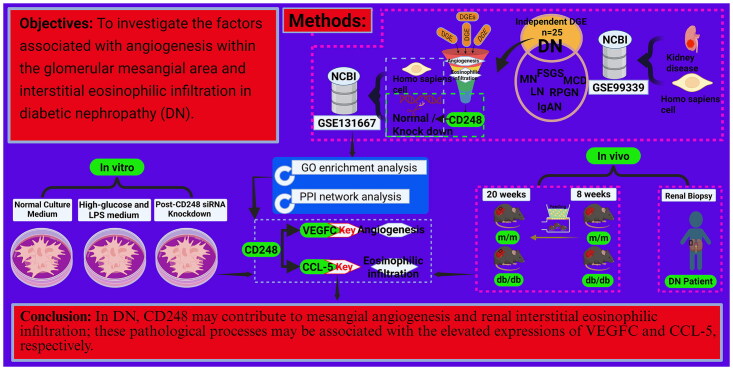

## 1. Introduction

Diabetic nephropathy (DN) is the most common chronic microvascular complication of diabetes mellitus, affecting approximately 30–40% of diabetic patients [1]; moreover, it is also a leading cause of end-stage renal disease [2]. In recent years, the incidence of DN has been gradually increasing, with prevalence rates ranging from approximately 17% to 35% in the United States and from approximately 29.6% to 49.6% in China [3]. Proteinuria and progressive renal function decline are the main features of DN, with pathological changes primarily occurring in the glomerulus. These changes are characterized by early thickening of the glomerular basement membrane and mesangial matrix proliferation, which can progress to glomerulosclerosis in later stages; moreover, these changes are often accompanied by tubular, interstitial, and vascular lesions [4]. A novel perspective focusing on the proximal tubules has been proposed; this notion involves early alterations in DN tubules that affect the interaction of the proximal tubules with the glomeruli, thus leading to renal dysfunction [5]. The pathogenesis of DN primarily involves oxidative stress, inflammation, podocyte injury, and activation of the renin-angiotensin-aldosterone system [6–10]. Increasing evidence suggests that renal inflammation promotes the onset and progression of DN, and a further understanding of immune-inflammatory pathways may offer new, more specific, and less toxic therapeutic targets for treating DN [11–13]. Three-dimensional imaging of DN renal biopsy specimens revealed the presence of isolated or clustered capillary-like structures around most glomeruli, thereby suggesting neovascularization around the glomeruli [14]. Reports have indicated the presence of vascular mesangial channels in the mesangial area of DN, with these channels being characterized by endothelial structures without a basement membrane, which has been demonstrated to represent neovascularization [15]. Additionally, studies have demonstrated more pronounced infiltration of eosinophils in DN patients, which indicates a possible correlation between glomerular neovascularization and eosinophilic infiltration in renal tissue, although the specific involved mechanisms remain unclear [14]. Previously, eosinophilic infiltration was believed to be linked to tubulointerstitial nephropathy; however, patients with DN and eosinophilic infiltration often do not benefit from treatment on the basis of this diagnosis in clinical practice. In this study, we defined eosinophil aggregation (IEA) as ≥5 eosinophils per high-power field (HPF) and used this criterion to assess eosinophilic infiltration.

CD248, which is a member of the C-type lectin-like domain family 14, plays a complex role in angiogenesis, with the potential to either promote or inhibit angiogenesis depending on specific contexts [16]. Clinical studies have suggested a potential role for CD248 in angiogenesis in kidney diseases [17]. CD248 is primarily expressed in the mesangial cells of healthy individuals and in mouse kidneys; moreover, its expression is upregulated in chronic kidney disease [18], and studies have suggested that its expression is induced by hypoxia and high levels of glycemic stress, with the potential involvement of hypoxia-inducible factors [19,20]. Therefore, our study aimed to investigate the role and mechanism of CD248 in vessel formation in the glomerular mesangial area and interstitial eosinophilic infiltration in DN, thereby providing insights into the pathological evolution of DN and offering new possibilities for the identification of effective therapeutic targets for DN.

## 2. Materials and methods

### 2.1. Bioinformatics analysis

#### 2.1.1. Identification of target genes associated with vessel formation and inflammatory cell chemotaxis in renal disease

We first identified DEGs specific to DN. The NCBI database was searched by using the term “kidney disease”; moreover, the criteria were established for human samples, and RNA-seq was used as the sample source, thus resulting in the GSE99339 dataset. The samples from DN, minimal change disease, focal segmental glomerulosclerosis, rapidly progressive glomerulonephritis, IgA nephropathy, membranous nephropathy, and lupus nephritis patients comprised the observation group, whereas the samples from individuals without kidney disease comprised the control group. The DEGs were searched using the Gene Expression Omnibus (GEO) online tool known as “Analyze with GEO2R” with *p* < 0.05 as the cutoff value; additionally, a Venn diagram was generated to calculate the set of independent DEGs of DN. The GeneCards website (https://www.genecards.org/) was subsequently used to search for NCBI gene summaries corresponding to the identified DEGs. The biological functions of the different DEGs were assessed to identify genes associated with vascular formation and inflammatory cell chemotaxis.

#### 2.1.2. Biological function analysis of CD248

The NCBI database was searched using the term “CD248”, with the criteria established for human cells and RNA-seq being used as the sample source, thereby resulting in the GSE131667 dataset. Cells with CD248 knockout were designated as the observation group, whereas cells without CD248 knockout served as the control group. The “Analyze with GEO2R” online tool in the GEO was used to identify DEGs with a significance level of *p* < 0.05 and logFC = 1. Enrichment analysis of the DEGs was conducted using the DAVID online tool (https://david.ncifcrf.gov/), with a focus on Gene Ontology (GO) annotations related to vessel formation and inflammation. DEGs associated with these GO terms were subjected to protein-protein interaction network analysis.

### 2.2. Human tissues

With informed consent, patients diagnosed with DN (*n* = 10, with 5 DN patients exhibiting renal failure and 5 DN patients exhibiting nonrenal failure), IgA nephropathy (*n* = 10), membranous nephropathy (*n* = 10), and minimal change disease (*n* = 10) *via* renal biopsies between July 1, 2023, and January 31, 2024, were subjected to CD248 immunohistochemical staining and multi-staining immunofluorescence colocalization assay.

### 2.3. Animals

#### 2.3.1. Experimental program

Eight-week-old male db/db mice (BKS. Cg-Dock7m +/+ Leprdb/Nju) and male m/m mice were purchased from Jiangsu Jicui Pharmatech Biotechnology Co., Ltd. (GemPharmatech Co., Ltd.), and all of the mice were housed in the Animal Experimentation Center of the Clinical Research Institute of the China-Japan Friendship Hospital. The experimental protocol was approved by the Animal Ethics Committee of the Clinical Research Institute (approval number: 2018-45-K34). The mice were divided into a control group (m/m, *n* = 6) and a model group (db/db, *n* = 6) and reared for 12 weeks before being removed for testing.

#### 2.3.2. Detection of indicators

The body weight of each group of mice was measured on a weekly basis, and the feeding, mental state, hair color, and activity characteristics of the mice were observed. Urine microalbumin (u-mAlb), the glomerular filtration rate (GFR), and fasting blood glucose (FBG) were measured before and after the experiment. This portion of this research has been previously published [21].

### 2.4. Cell culture and experimental interventions

#### 2.4.1. Mesangial cell culture

The murine glomerulus-derived mesangial cell line SV40 MES 13 (CRL-1927; ATCC, USA) was used for the experiments. The cells were maintained under standard culture conditions (37 °C, 5% CO_2_) in SV40 MES 13-specific growth medium (C5523-500; BD Biosciences, USA). At five passages after subculture, transient transfection was performed to knock down CD248 expression *via* siRNA interference.

#### 2.4.2. CD248 silencing in mesangial cells

The siRNA sequence targeting CD248 (sense: 5′-ACACCGCCUUCACCAACUTT-3′; antisense: 5′-AGUUGGUGAAGGCGGUGUCTT-3′) was synthesized by GenePharma (Shanghai GenePharma Co., Ltd., Shanghai, China). The transfection procedures were performed as follows. First, cells were seeded in plates 24 h before transfection (24-well plate, 8*10^4^ cells/well; 6-well plate, 3.8*10^5^ cells/well). The cells were cultured overnight, and transfection was initiated when the cell confluence reached approximately 60%. Preparation of the siRNA transfection complex was completed using the following steps (24-well plate example). 1. siRNA mixture: 8.5 μL of buffer was aliquoted into a sterile RNase-free EP tube. Afterwards, 15 pmol of siRNA was added and thoroughly mixed *via* pipetting. 2. siRNA/PLUS complex: Three microliters of PLUS Transfection Reagent was added to each premix tube. Immediate vigorous pipetting (>10 mixing cycles) ensured homogeneous complex formation. The siRNA/PLUS complex was subsequently added to the culture medium. After 8 h of culture, the transfection medium was replaced with high-glucose and lipopolysaccharide (LPS)-containing medium (30 mmol/L glucose and 20 μg/ml LPS) for subsequent stimulation. Transfection efficiency was validated *via* fluorescent-labeled siRNA tracking and WB analysis of CD248 protein expression.

#### 2.4.3. Cell immunofluorescence staining

Glass coverslips (14 mm in diameter) were sterilized *via* immersion in 75% ethanol for 15 min and placed into a 24-well plate. Mesangial cells were seeded at a density of 8*10^4^ cells per well, transfected, and subjected to the appropriate treatments for 48 h. The culture medium was then removed, and the cells were gently washed three times with ice-cold phosphate-buffered saline (PBS) (5 min per wash). The cells were fixed with 4% paraformaldehyde at room temperature (RT) for 20 min, followed by permeabilization with 0.1% Triton X-100 for 15 min. Nonspecific binding sites were blocked with 5% goat serum at RT for 30 min. The cells were incubated with primary antibodies at 4 °C overnight and then washed three times with PBS (5 min per wash). Fluorescently labeled secondary antibodies were applied, and the samples were incubated for 1 h at RT in the dark. Nuclei were counterstained with 4′,6-diamidino-2-phenylindole (DAPI) for 5 min. Coverslips were mounted with antifade mounting medium and imaged with a laser scanning confocal microscope.

### 2.5. Immunohistochemical staining

Renal tissue was sectioned in paraffin blocks at a thickness of 2 µm, dewaxed in water and antigenically repaired with pepsin at 37 °C for 40 min. Endogenous peroxidase was removed with H2O2 treatment for 10 min at room temperature, after which the samples were washed three times with PBS for 5 min/session, blocked with sheep serum antibody for 30 min, titrated with primary antibodies (anti-CD248 antibody, 1:500, 60170-1-Ig, Proteintech, China; VEGFC polyclonal antibody, 1:400, 22601-1-AP, Proteintech; CCL-5 polyclonal antibody, 1:200, 12000-1-AP, Proteintech; anti-CD3 antibody, 1:150, ab135372, Abcam, UK; anti-CD31 antibody, 1:2000, ab182981, Abcam), and incubated overnight at 4 °C. The sections were removed and rewarmed at room temperature for 1 h, and the corresponding secondary antibody was added dropwise and incubated at room temperature for 30 min. DAB color developer was added for 5–10 min, and the reaction was terminated with hydrogen peroxide. Hematoxylin was used for nuclear staining for 1 min, followed by gradient ethanol dehydration, xylene clearing, and the addition of a drop of neutral gelatin film. The stained sections were observed under a microscope and photographed.

### 2.6. Multiple immunofluorescence staining

Pepsin was used for antigen repair for 40 min at 37 °C; additionally, Triton X-100 was used for permeabilization for 10 min, and the sheep serum antibody was blocked for 30 min. Primary antibodies were added (CD248 was coincubated with VEGFC antibody, and CD248 was coincubated with CCL-5 antibody, all of which were diluted at a ratio of 1:100) and incubated at 4 °C overnight. Finally, FITC and Cy3 were added and incubated at 37 °C in the dark for 1 h. The slides were sealed and observed by fluorescence microscopy.

### 2.7. Western bblotting analysis

Total protein was extracted from fresh tissues or cultured cells using RIPA lysis buffer (PC101, Epizyme, China) supplemented with a protease and phosphatase inhibitor cocktail (GRF103, Epizyme, China). Total protein lysate (30 μg) was separated *via* 6% or 10% sodium dodecyl sulfate-polyacrylamide gel electrophoresis and transferred onto polyvinylidene difluoride membranes (WJ002, Epizyme, China). The membranes were blocked with rapid blocking buffer (PS108P, Epizyme, China) for 15 min and then incubated overnight at 4 °C with primary antibodies (details provided in the reagent list in Supplemental Table 1). The membranes were subsequently incubated with the corresponding secondary antibodies at room temperature for 1 h. The immunoreactive bands were visualized using an enhanced chemiluminescence detection system (Thermo Fisher Scientific, USA).

### 2.8. Statistical analyses

SPSS 26.0 was used for the statistical analyses. For the continuous variables, data that conformed to a normal distribution are expressed as the mean ± standard deviation, and a t test was applied for the comparisons. Continuous variables that did not conform to a normal distribution are expressed as medians and interquartile intervals and were compared using the rank sum test. R 4.3.2 was used to produce the Venn diagrams for the DEGs of the different diseases. ImageJ software was used for immunofluorescence colocalization analysis, and GraphPad Prism 10.0 was used to visualize the immunofluorescence colocalization data.

## 3. Results

### 3.1. Bioinformatics analysis

#### 3.1.1. Identification of target genes involved in vessel formation and the chemotaxis of inflammatory cells

**Figure 1.**
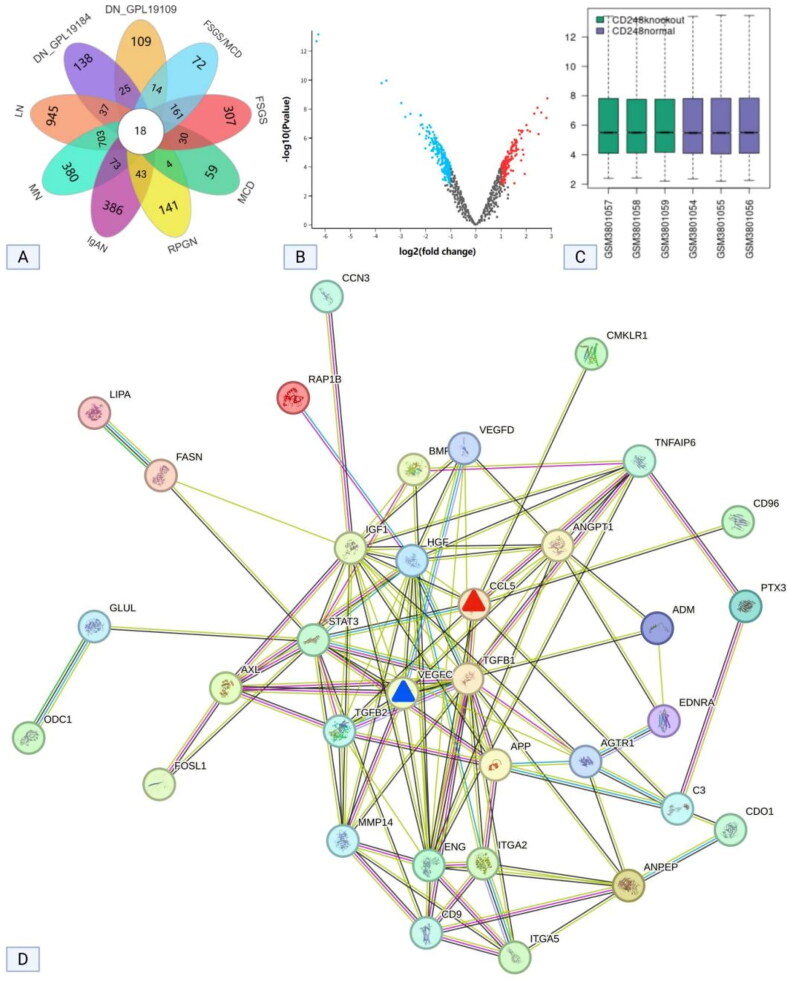
Results of the bioinformatic analysis. (A) Venn diagram of DEGs across various diseases (dataset GSE99339). (B) The volcano plot of the 391 identified DEGs, with red indicating upregulation of DGE following CD248 knockout and blue indicating downregulation (dataset GSE131667). (C) The box plots of the DEGs demonstrate comparable levels between CD248 knockout and non-knockout samples, indicating their comparability (dataset GSE131667). (D) The protein-protein interaction network analysis. The results showed that VEGFC and CCL-5 were all key proteins (marked by blue and red triangular symbols), potentially involved in CD248-mediated mesangial vascular formation and eosinophil infiltration in the interstitium (dataset GSE131667). DN, diabetic nephropathy; DN_GPL19184, DN data from platform GPL19184; DN_GPL19109, DN data from platform GPL19109; FSGS/MCD, focal segmental glomerulosclerosis or minimal change disease; FSGS, focal segmental glomerulosclerosis; MCD, minimal change disease; IgAN, IgA nephropathy; MN, membranous nephropathy; RPGN, rapidly progressive glomerulonephritis; LN, lupus nephritis; DEGs, differentially expressed genes; VEGFC, vascular endothelial growth factor C; CCL5, C-C motif chemokine ligand 5.

Different disease-specific DEGs were filtered based on the screening criteria (see additional file 1). A Venn diagram analysis was performed to identify the unique set of DEGs specific to DN (Figure 1(A)), thus resulting in 25 unique DEGs, with the gene IDs listed as follows: 10513, 347733, 2328, 1998, 1027, 23014, 1906, 56934, 11168, 56890, 8490, 57124, 54619, 9364, 653784, 51280, 55816, 286527, 8639, 7033, 51066, 4603, 5029, 10516, and 113146. According to the NCBI gene summaries, only CD248 was observed to be simultaneously associated with vascular formation and inflammatory cell chemotaxis.

#### 3.1.2. Biological function analysis of CD248

Cells with CD248 knockout were designated as the observation group, whereas cells without CD248 knockout served as the control group. A total of 391 DEGs were identified based on the criteria of *p* < 0.05 and logFC = 1 (additional file 2). Volcano plots and box plots are depicted in Figure 1(B) and (C).

The 391 DEGs were subjected to GO enrichment analysis utilizing functional annotation information from the GO database (the results are provided in additional file 2). Based on the annotation information, GO terms including GO:0006954 inflammatory response, GO:0001525 angiogenesis, GO:0097084 vascular smooth muscle cell development, and GO:0031093 platelet alpha granule lumen were selected, thus indicating their relevance to vascular formation and inflammation, which potentially contributes to vascular formation and eosinophilic infiltration. Protein-protein interaction network analysis was conducted on the DEGs associated with these four GO processes (Figure 1(D)). The results revealed that VEGFC played a key role in the promotion of vessel formation *via* CD248. Moreover, CD248 mainly promoted eosinophilic infiltration *via* CCL-5, which is an eosinophil chemoattractant.

### 3.2. Immunohistochemical staining and immunofluorescence colocalization assay of human renal tissue

**Figure 2.**
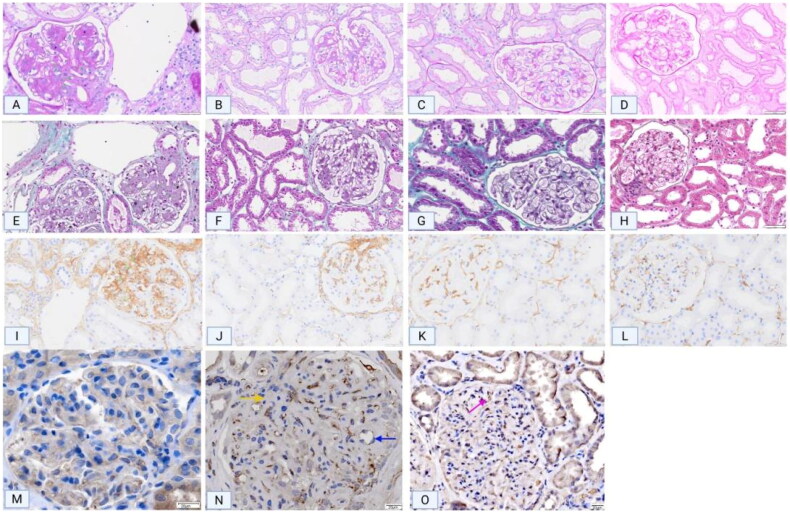
PAS, masson, and IHC staining of CD31, VEGFC, and CD248 in renal tissues of glomerular disease patients. (A, E, I) Pronounced CD248 immunostaining and vascular formation in the mesangial area of DN. In IgAN (B, F, J), MN (C, G, K), and MCD (D, H, L), CD248 expression is significantly attenuated compared to DN, accompanied by reduced mesangial vascular formation. (M) Absence of CD31 signal in the mesangial area of adult paracancerous tissues. (N) CD31-positive vascular channels within the mesangial area in DN (yellow arrows: glomerular vascular pole; blue arrows: mesangial neovascularisation). (O) VEGFC expression in DN. VEGFC IHC staining shows positivity in the mesangial area (purple arrows). A-D: PAS staining. E-H: Masson trichrome staining. I-L: CD248 IHC staining. M-N: CD31 IHC staining. PAS, periodic Acid-Schiff; IHC, immunohistochemistry.

**Figure 3.**
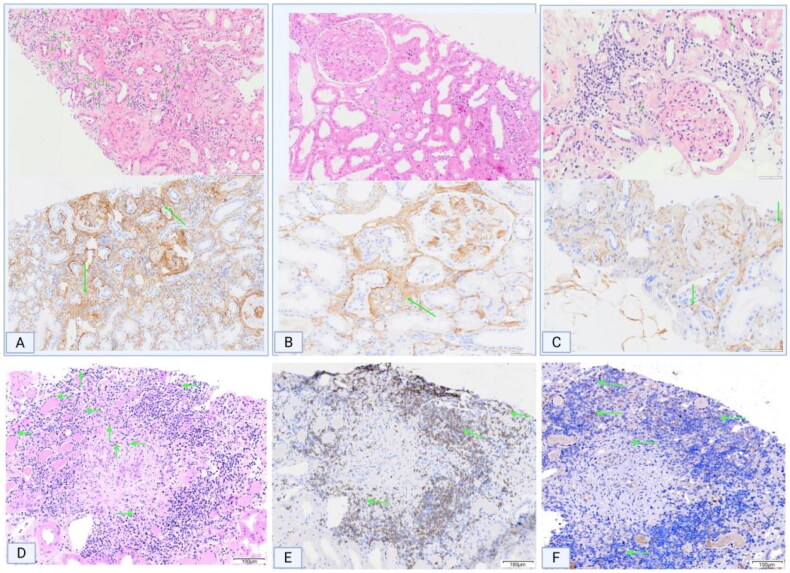
Immunostaining of CD3, CCL5, and CD248 in renal tissues. (A-C) CD248 staining and eosinophil infiltration in DN patients. The data demonstrate a gradual decrease in CD248 expression levels (green arrows), which correlates with a progressive reduction in eosinophil infiltration at the same anatomical sites (green arrows). (D-F) Serial sections of DN renal tissues reveal that regions with prominent eosinophil infiltration are frequently co-localized with CD3-positive T lymphocyte infiltration. Furthermore, these areas exhibit marked positivity for CCL5 staining (green arrows).

**Figure 4.**
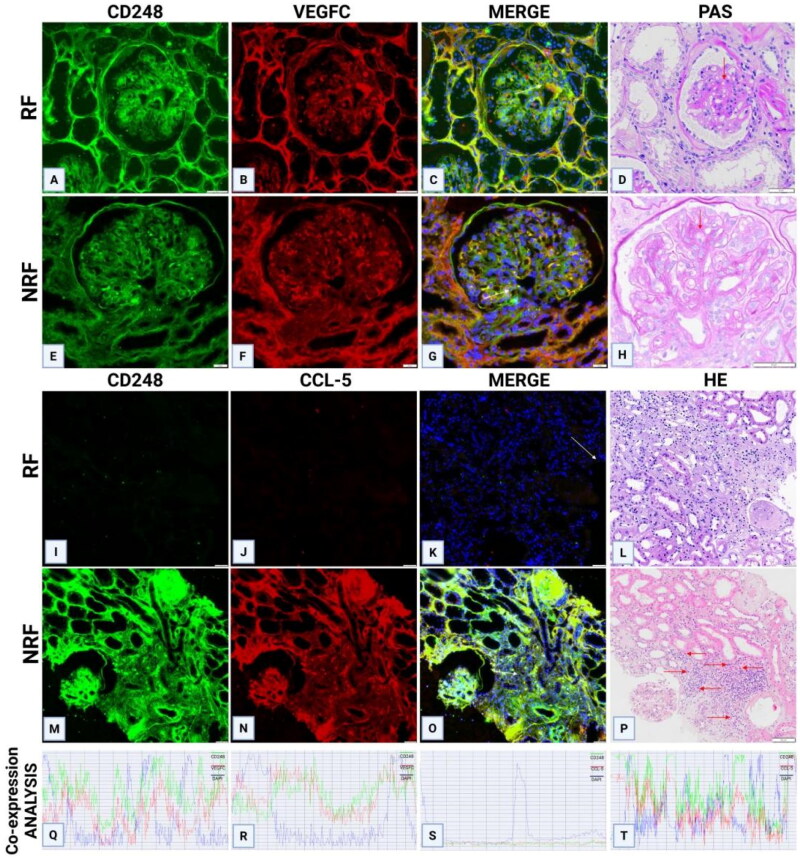
IF co-localisation of CD248 with VEGFC or CCL5 in renal tissues of DN patients with/without RF. (A-H) In both RF and NRF DN patients, IF staining revealed co-expression of CD248 and VEGFC in renal tissues, with overlapping signals localized to regions of neovascularisation (red arrows). (I-L) In RF-DN patients with no evidence of interstitial eosinophilic infiltration, IF signals for CD248 and CCL5 were undetectable. (M-P) In contrast, NRF-DN patients with prominent eosinophilic infiltration (red arrows) exhibited robust co-localisation of CD248 and CCL5. (Q-T) Co-localisation analysis was performed on merged immunofluorescence images (panels C, G, K, O; white arrows in merged images). D, H: PAS staining; L, P: HE staining. IF, immunofluorescence; RF, renal failure; NRF, non-renal failure; HE, hematoxylin and eosin.

**Figure 5.**
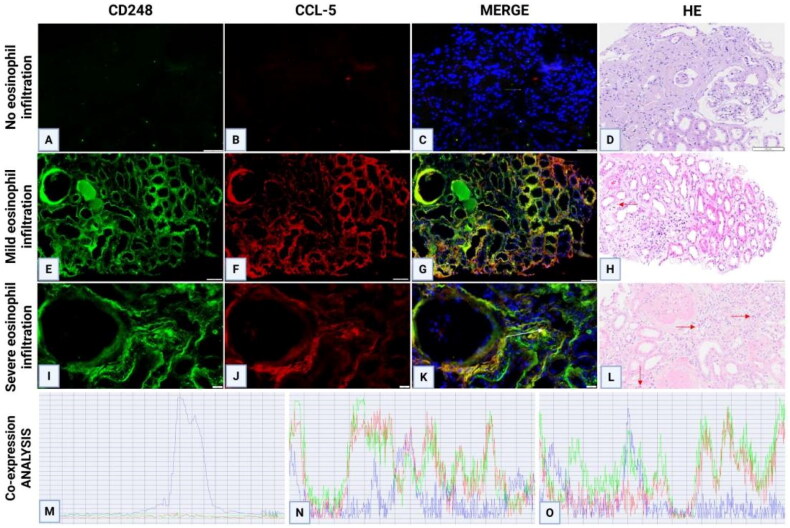
IF co-localization staining of CD248 and CCL5 in renal tissues of DN patients with varying degrees of eosinophilic infiltration. (A–L) IF analysis showed a progressive enhancement of CD248 and CCL5 expression correlating with elevated eosinophilic infiltration in the renal interstitium (red arrows). (M–O) Co-localization analysis of merged images revealed significant spatial overlap between CD248 and CCL5 signals. Eosinophilic infiltration grading criteria: None, No eosinophils detected in the renal interstitium; mild: <5 eosinophils/HPF; severe, >10 eosinophils/HPF. HPF, high-power field.

Immunohistochemical staining of CD248 revealed high expression in the mesangial area of DN tissues, whereas significantly reduced expression was observed in other kidney diseases. The expression pattern of CD248 corresponded to the location of blood vessels within the mesangial area (Figure 2(A–L)). To confirm the notion that channels in the mesangial area represent newly formed blood vessels, CD31 staining (a widely accepted marker for vascular endothelial cells) was performed, and the results were compared between the paracancerous tissues of adult kidneys and the renal tissues of DN patients. CD31 staining was observed to be negative in the mesangial area of adult paracancerous tissues, whereas channels in the mesangial area of DN tissues were positive for CD31 staining. Furthermore, VEGFC staining of DN renal tissues revealed positive staining in the mesangial area (Figure 2(M–O)). Immunohistochemical quantification revealed a positive correlation between CD248 expression levels and the density of eosinophilic infiltration (Figure 3(A–C)). Serial sections of DN renal tissues revealed that regions with marked eosinophilic infiltration were often accompanied by T lymphocyte infiltration. CCL-5 staining in these areas also revealed strong positivity, thereby suggesting that T lymphocytes may secrete the eosinophil chemokine CCL-5, which would lead to eosinophilic infiltration in the renal interstitium (Figure 3(D–F)). Moreover, CD248 and VEGFC were coexpressed in the glomerular mesangial area of renal tissues in both renal failure and nonrenal failure DN patients, and these expression patterns were consistent with those in the neovascular area (Figure 4(A–H)). Immunofluorescence staining of renal tissues from DN patients revealed that the expression of CD248 and CCL-5 was mainly associated with eosinophils, with no significant correlation being observed with renal function (Figure 41–R); furthermore, as the degree of eosinophilic infiltration increased, the expression of CD248 and CCL5 also increased. Immunofluorescence colocalization analysis revealed coexpression of CD248 and CCL-5 (Figure 5).

### 3.3. Animal experiments

#### 3.3.1. Detection indicators

The FBG, u-mAlb, and GFR values of the two groups of mice at 8 and 20 weeks of age are shown in Table 1. We present the pathological scoring of renal tubulointerstitial and glomerular lesions and quantification of tubular cell necrosis in the Supplemental Table 2.

**Table 1.** Fasting blood glucose, urinary microalbumin, and glomerular filtration rate in 8 and 20-week-old mice.

	8-week-old mice	20-week-old mice
FBG (mmol/L)		
db/db	11.5 ± 3.7	25.9 ± 6.5*
m/m	5.1 ± 1.3	7.0 ± 0.4
u-mAlb (mg/12h)		
db/db	0.020 ± 0.008	0.060 ± 0.014*
m/m	0.010 ± 0.008	0.010 ± 0.001
GFR (μl/min)		
db/db	471.7 ± 75.8	774.1 ± 90.3
m/m	250.3 ± 60.7	209.1 ± 37.4

FBG, fasting blood glucose; GFR, glomerular filtration rate; u-mAlb, urine microalbumin (obtained for 12 h of urine collection).

* Compared to m/m, *p* < 0.05.

#### 3.3.2. Neovascularization in the glomerular mesangial area of db/db mice

**Figure 6.**
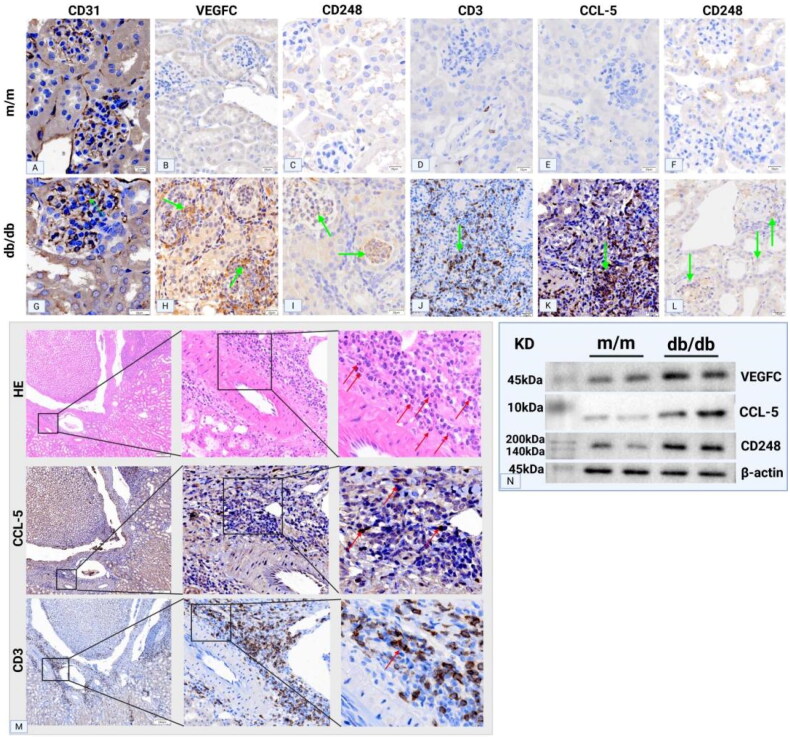
IHC staining results in the renal tissues of db/db DN mouse models. (A-C) In m/m mice, glomerular staining for CD248, VEGFC, and CD31 was negative. (D-F) Similarly, renal interstitial staining for CD248, CCL-5, and CD3 in m/m mice was negative. (G-I) In db/db mice: Glomeruli: CD31 staining revealed newly formed vascular channels in the mesangial area (green arrows), accompanied by increased expression of VEGFC and CD248 in the mesangial region. (J-L) In db/db mice: Renal interstitium: Compared to m/m mice, CD248 and CCL-5 expression was elevated (green arrows), with significantly enhanced infiltration of CD3-positive T lymphocytes. (M) Serial sections further revealed that areas of eosinophil aggregation coincided with intensified CD3-positive T lymphocyte infiltration and enhanced CCL-5 expression (red arrows). (N) WB analysis confirmed higher expression levels of CD248, CCL-5, and VEGFC in renal tissues of db/db mice compared to m/m mice. WB, Western blot.

CD31 immunohistochemical staining in db/db mice revealed neovascularization in the mesangial area (Figure 6(G)), which was accompanied by increased expression of VEGFC and CD248 in the mesangial region (Figure 6(H) and (I)). In contrast, this phenomenon was not observed in the renal tissues of m/m mice (Figure 6(A–C)).

#### 3.3.3. Eosinophilic infiltration in the renal interstitium of db/db mice

Focal eosinophilic infiltration was observed in the renal interstitium of db/db mice. Serial sections of renal tissues revealed that areas with marked eosinophilic infiltration exhibited aggravated infiltration of CD3-positive T lymphocytes and increased expression of CCL-5, which is a chemokine exhibiting eosinophil chemotactic functions that is secreted by T lymphocytes (Figure 6(M)). Compared with m/m mice (Figure 6(D–F)), db/db mice exhibited increased expression of CD248 and CCL-5 in the renal interstitium, along with significantly increased infiltration of CD3-positive T lymphocytes (Figure 6(J–L)). WB analysis further confirmed the elevated expression of CD248, CCL-5, and VEGFC in the renal tissues of db/db mice compared with m/m mice (Figure 6(N)).

#### 3.3.4. Increased CD248 in the renal tissues of db/db mice, accompanied by elevated expression of CCL-5 and VEGFC

**Figure 7.**
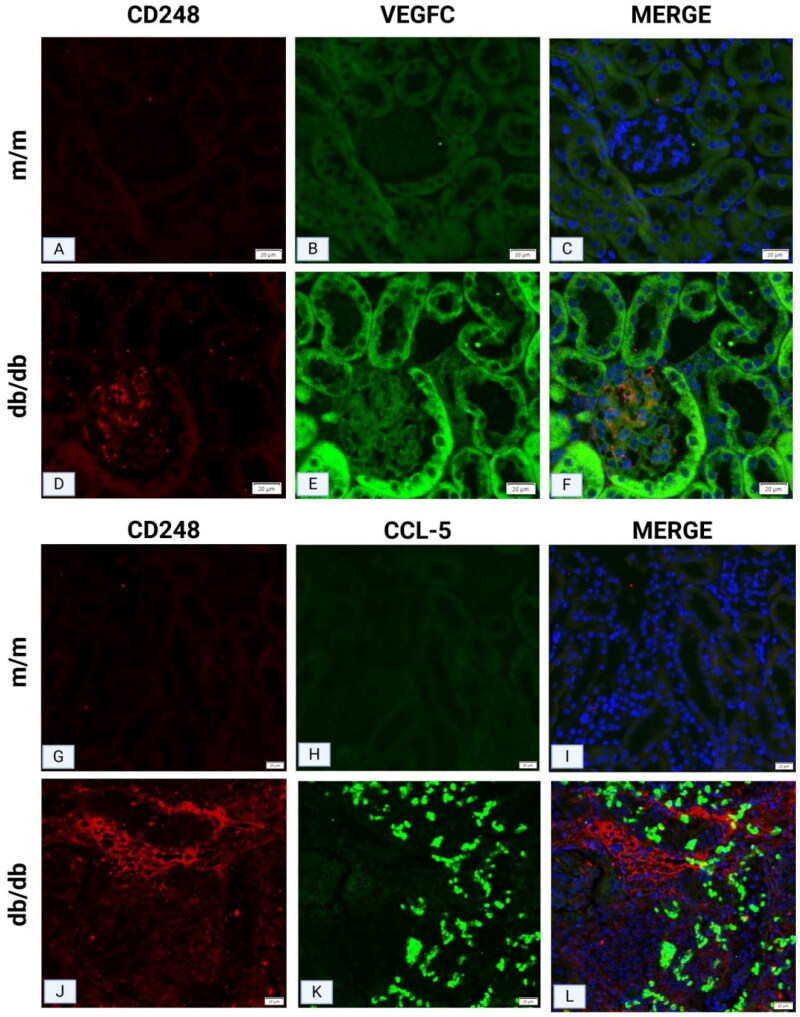
Multiplex IF staining of CD248 with CCL-5 and VEGFC in mouse renal tissues. (A-C) IF staining of CD248 and VEGFC in renal tissues of mice in the m/m group was negative. (D-F) In the db/db group, both CD248 and VEGFC expressions were increased in glomeruli, with vascular channels in the mesangial area. (G-I) IF staining of CD248 and CCL-5 in the renal interstitium of m/m mice was negative. (J-L) Increased CD248 expression in the interstitium of db/db mice was accompanied by elevated CCL-5 expression.

Multiplex immunofluorescence staining of CD248, VEGFC, and CCL-5 was performed on mouse renal tissues. In the renal tissues of the m/m group, immunofluorescence staining for CD248 and VEGFC was negative (Figure 7(A–C)). In contrast, db/db mice exhibited increased expression of both CD248 and VEGFC in the glomeruli, which was accompanied by neovascularization in the mesangial area (Figure 7(D–F)), thereby suggesting a potential correlation between CD248 and VEGFC in collectively promoting neovascularization in the mesangial region. In the renal interstitium, immunofluorescence signals for CD248 and CCL-5 were negative in m/m mice (Figure 7(G–1)). However, in db/db mice, increased expression of CD248 in the interstitium was associated with elevated CCL-5 expression (Figure 7(J–L)), thus indicating a potential interaction between CD248 and CCL-5 in promoting eosinophilic infiltration within the renal interstitium.

### 3.4. *In vitro* experiments

#### 3.4.1. High glucose conditions and LPS induce elevated CD248 expression in mesangial cells

**Figure 8.**
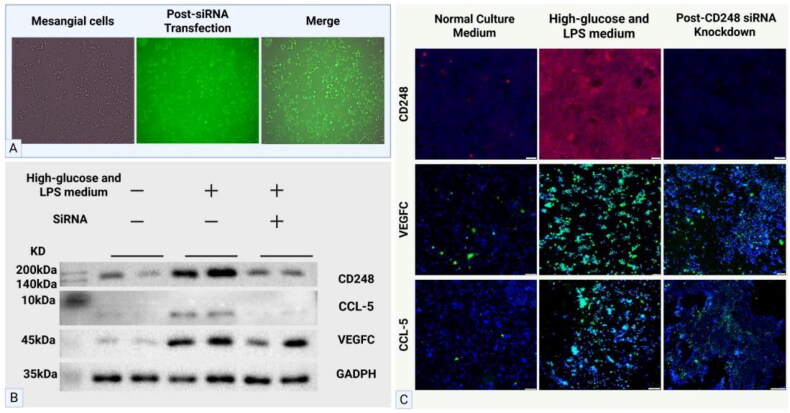
The effects of CD248 knockdown in mesangial cells on VEGFC and CCL-5 expression. (A) FITC-labeled siRNA transfection in mesangial cells was observed under fluorescence microscopy, showing a transfection efficiency exceeding 90%. (B) WB analysis confirmed high-glucose and LPS stimulation increased CCL-5 and VEGFC expression in mesangial cells, which was attenuated by CD248 knockdown. (C) Mesangial cells cultured in high-glucose and LPS medium exhibited significantly elevated CD248 expression compared to those in normal culture conditions (where CD248 was almost undetectable). CD248 knockdown markedly suppressed the high-glucose and LPS-induced upregulation of CD248 expression. The expression levels of VEGFC and CCL-5 were significantly upregulated under high-glucose and LPS stimulation. Following CD248 knockdown, the protein expression of both VEGFC and CCL-5 exhibited marked suppression.

FITC-labeled siRNA was transfected into mesangial cells. When 80,000 cells per well were seeded in 24-well plates and cultured at 37 °C in a 5% CO_2_ incubator for approximately 30 h, the mesangial cells adhered well, with cell confluence reaching 60%. At 8 h after transfection with fluorescently labeled siRNA, fluorescence microscopy revealed that the transfection efficiency exceeded 90% (Figure 8(A)). Compared with normal culture conditions, the stimulation of mesangial cells with culture medium containing high glucose and LPS resulted in significantly increased CD248 expression compared with cells under normal culture conditions, in which CD248 was barely detectable. After transfection with CD248-knockdown siRNA followed by stimulation with high-glucose and LPS-containing medium, CD248 expression was markedly reduced, and VEGFC and CCL-5 expression was also decreased, compared with that in high-glucose and LPS-stimulated conditions without knockdown (Figure 8(C)).

#### 3.4.2. CD248 knockdown reduces CCL-5 and VEGFC expression in mesangial cells under high-glucose and LPS conditions

CD248-knockdown and nonknockdown mesangial cells were cultured in six-well plates containing either normal medium or high-glucose and LPS medium and divided into the following three groups: the normal medium group, the high-glucose and LPS medium group, and the high-glucose and LPS medium + siRNA group. WB analysis revealed that high-glucose and LPS stimulation upregulated CCL-5 and VEGFC expression in mesangial cells, whereas CD248 knockdown attenuated this effect (Figure 8(B)).

## 4. Discussion

In DN, renal histopathology, mesangial angiogenesis and renal interstitial eosinophilic infiltration are observed. To elucidate their mechanisms, this study initially identified differential genes associated with DN-related angiogenesis and inflammatory cell recruitment. CD248 was observed to interact with both angiogenesis and inflammatory cell chemotaxis, potentially *via* VEGFC and CCL-5. To validate the bioinformatics analysis results, immunohistochemistry, immunofluorescence, and WB were performed on adjacent nontumor renal tissues, DN patient tissues, and db/db DN mouse renal tissues. CD248 was knocked down in mesangial cells *via* siRNA to investigate its regulatory effects on VEGFC and CCL-5. In human kidney tissues, more channels were observed in the mesangial area of DN patients compared with patients with IgA nephropathy, membranous nephropathy, or minimal change nephropathy. CD31 staining revealed that these channels contained endothelial cells that formed a vascular structure, and increased expression of CD248 and VEGFC was observed in the mesangial area. Regions of intense renal interstitial eosinophilic infiltration were correlated with elevated CD248 expression, increased CD3-positive T lymphocyte infiltration, and increased CCL-5 levels. Similar findings were observed in db/db mice, with WB confirming increased CD248, VEGFC, and CCL-5 levels compared with those in m/m controls. Moreover, siRNA-mediated CD248 silencing in mesangial cells suppressed high-glucose- and LPS-induced VEGFC and CCL-5 expression, thus supporting their mechanistic link.

DN is a microvascular complication caused by diabetes mellitus. Diabetes induces tissue ischemia and hypoxia, which strongly stimulate the formation of both large and small blood vessels [22]. Historically, research on neovascularization in diabetic complications has primarily focused on diabetic retinopathy [23]. However, recent studies have demonstrated that neovascularization also occurs in DN. In the early stages of DN, hypoxia induces the generation of hypoxia-inducible factors and endothelial growth factors to maintain renal vascular density, thereby leading to increased angiogenesis and immature vessel development. In the later stages, renal hypoxia worsens, and inflammatory reactions intensify, thus leading to abnormal angiogenesis and vascular leakage. Additionally, several studies have suggested that many vascular growth factors are associated with renal development and glomerular filtration functions [24,25]. Our research also demonstrated the presence of neovascularization in the renal tissues of DN patients, which is consistent with previous findings.

Eosinophilic infiltration in chronic kidney disease (CKD) has been reported on multiple occasions. Clinical studies have indicated that eosinophil aggregates are observed in the renal interstitium of 17% of CKD patients, with DN being more prevalent (50%) than IgA nephropathy, membranous nephropathy, and mesangial proliferative glomerulonephritis. In DN patients, the presence of eosinophilic aggregates in renal tissues is correlated with GFR, proteinuria, hematuria, glycated hemoglobin, blood eosinophil count, tubular injury, and chronic interstitial inflammation. Additionally, eosinophilic aggregation predicts poorer renal prognosis, with steroid/immunosuppressive therapy demonstrating no significant benefits to renal outcomes [26–29]. Our study also revealed a significant presence of eosinophilic aggregates in DN renal tissues. However, the pathways of eosinophil migration and their impact on prognosis remain inconsistent, and further investigations into related mechanisms are needed.

CD248 is a type I transmembrane glycoprotein that was initially identified in tumor vascular endothelial cells [30]. In healthy adults, CD248 appears to be expressed only in perivascular cells in some lung vessels, the spleen, and the endometrium, as well as in mesangial cells in renal glomeruli [31]. In diseases, CD248 may serve as a prognostic factor for tumors and is upregulated in inflammatory states [32]. It also promotes angiogenesis in various tissues or diseases, such as skeletal muscle and lung cancer [33,34]. Recent studies have highlighted the significant role of CD248 in renal diseases. Smith et al. demonstrated CD248 expression in the mesangial cells of renal glomeruli in normal kidney tissues. Moreover, they reported increased CD248 expression in IgA nephropathy, with higher expression levels observed in the later stages of the disease; additionally, clinical follow-up indicated that CD248 is an independent predictor of renal survival [18]. Animal experiments have demonstrated that the knockout of CD248 improves glomerular dysfunction associated with DN [20]. Clinical studies have indicated that CD248 overexpression in the vasculature of renal cell carcinoma patients predicts adverse clinical outcomes [17,35]. Our study revealed significantly increased expression of CD248 in DN renal tissues compared with that in patients with IgA nephropathy, membranous nephropathy, and minimal change disease, which is possibly related to vascular formation in DN patients. Additionally, our research revealed a correlation between CD248 expression levels and eosinophil aggregation in DN.

We have preliminarily explored the potential mechanisms by which CD248 promotes vascular formation and eosinophil aggregation in DN. VEGFC is a key participant in VEGF signaling and is crucially involved in the development and remodeling of lymphatic and vascular systems [36,37]. Studies have indicated that inflammation can induce the upregulation of VEGFC expression [38–40]. Bioinformatics analysis suggested that CD248 may influence the development of neovascularization in the renal tissue of DN patients by modulating VEGFC. Additionally, CCL-5 is a chemotactic factor associated with antigen presentation, leukocyte activation and survival, chronic inflammation, and angiogenesis [41,42]. The STRING database describes CCL-5 as a chemotactic agent for monocytes, memory helper T cells, and eosinophils. To our knowledge, this is the first study reporting a positive correlation between CD248 and eosinophil aggregation in renal tissues. The limitation of this study is that CD248 knockdown was not tested *in vivo* to assess its effects on mesangial angiogenesis or renal interstitial eosinophilic infiltration in DN models. Therefore, further related research is anticipated.

## 5. Conclusion

In DN, CD248 may contribute to mesangial angiogenesis and renal interstitial eosinophilic infiltration; moreover, these pathological processes may be associated with elevated expression of VEGFC and CCL-5, respectively.

## Supplementary Material

Supplemental Material

Additional file 2.xlsx

Additional file 1.xlsx

## Data Availability

The data presented in this study can be found in the corresponding author.
