# Supplementary material for: Mesangial angiogenesis and interstitial eosinophilic infiltration in diabetic nephropathy are associated with elevated CD248 expression
Source: Ren Fail. 2025 Jun 1;47(1):2510552. doi: 10.1080/0886022X.2025.2510552 (PMC12128132; doi:10.1080/0886022X.2025.2510552)
Supplement: Supplemental Material [file IRNF_A_2510552_SM9005.docx]

**Supplemental Table 1.** Information on chemicals and reagents.

| Antibody | Supplier | Catalog number | Dilution ratio |
| --- | --- | --- | --- |
| Anti-CD248 antibody | Proteintech (China) | 60170-1-Ig | IF (1:100), IHC (1:500), WB (1:2000). |
| VEGF-C Polyclonal antibody | Proteintech (China) | 22601-1-AP | IF (1:100), IHC (1:400), WB (1:1000). |
| CCL-5 Polyclonal antibody | Proteintech (China) | 12000-1-AP | IF (1:100), IHC (1:200), WB (1:800). |
| Anti-CD3 antibody | Abcam (United Kingdom) | Ab135372 | IHC (1:150) |
| Anti-CD31 antibody | Abcam (United Kingdom) | Ab182981 | IHC (1:2000) |
| FITC-conjugated Goat Anti-Rabbit IgG | Proteintech (China) | SA00003-2 | IF (1:200) |
| Cy3-conjugated Rabbit Anti-Mouse IgG | Proteintech (China) | SA00009-1 | IF (1:200) |
| SiRNA | GenePharma (China) |  | 15 pmol/well (24-well plate)  75 pmol/well (6-well plate) |
| Protein Loading Buffer | Solarbio (China) | P1015 | WB (1:3) |
| TritonX-100 | Sigma-Aldrich (United States of America) | T8787-50ML | IF (1:1000) |
| SV40 MES 13 Medium | BD (United States of America) | C5523-500 |  |
| RIPA Lysis Buffer | Epizyme (China) | PC101 |  |
| Protease and Phosphatase Inhibitor Cocktail | Epizyme (China) | GRF103 |  |
| Rapid Blocking Buffer | Epizyme (China) | PS108P |  |
| Rapid Electrophoresis Buffer | APPLYGEN (China) | B2005 |  |
| Rapid Transfer Buffer | APPLYGEN (China) | B2006 |  |

IF, Immunofluorescence; IHC, Immunohistochemistry; WB, Western Blot.

**Supplemental Table 2.** Pathological Scoring of Renal Tubulointerstitial and Glomerular Lesions and Quantification of Tubular Cell Necrosis.

| Specimen Origin | Specimen ID | Tubular Atrophy ^1^ | Interstitial Fibrosis ^1^ | Arteriosclerosis ^1^ | Arteriolar Hyalinosis ^1^ | Number of Tubular Cell Necrosis ^1^ |
| --- | --- | --- | --- | --- | --- | --- |
| Human ^2^ | 2435866 | 2 | 2 | 2 | 1 | 0 |
|  | 2401472 | 1 | 1 | 2 | 1 | 0 |
|  | 2419290 | 1 | 1 | 2 | 1 | 0 |
|  | 2263935 | 2 | 2 | 2 | 1 | 0 |
|  | 1481018 | 1 | 1 | 2 | 1 | 0 |
|  | 2256635 | 2 | 2 | 2 | 0 | 0 |
|  | 2246108 | 2 | 2 | 2 | 1 | 0 |
|  | 2244482 | 2 | 2 | 2 | 1 | 0 |
|  | 2220825 | 2 | 2 | 2 | 1 | 0 |
|  | 1497166 | 1 | 1 | 2 | 1 | 0 |
| Mouse ^2^ | B1 | 1 | 1 | 0 | 0 | 0 |
|  | B2 | 1 | 1 | 1 | 0 | 0 |
|  | B3 | 1 | 1 | 1 | 0 | 0 |
|  | B4 | 1 | 1 | 0 | 0 | 0 |
|  | B5 | 1 | 1 | 1 | 1 | 0 |
|  | B6 | 1 | 1 | 0 | 0 | 0 |

^1^ Pathological scoring was based on the diabetic nephropathy (DN) classification criteria proposed by the International Society of Nephrology in 2010. (https://journals.lww.com/jasn/abstract/2010/04000/pathologic_classification_of_diabetic_nephropathy.7.aspx).

^2^ Human renal biopsy specimens were obtained from patients with confirmed DN, and kidney tissues were harvested from db/db mice, a well-established murine model of DN.
